# Community Fire Risk Reduction: Longitudinal Assessment for HomeSafe Fire Prevention Program in Canada

**DOI:** 10.3390/ijerph20146369

**Published:** 2023-07-15

**Authors:** Samar Al-Hajj, Larry Thomas, Shelley Morris, Joseph Clare, Charles Jennings, Chris Biantoro, Len Garis, Ian Pike

**Affiliations:** 1 Department of Epidemiology and Population Health, Faculty of Health Sciences, American University of Beirut, Beirut 11-0236, Lebanon; 2 British Columbia Injury Research and Prevention Unit, British Columbia Children’s Hospital Research Institute, Vancouver, BC V6H 3V4, Canada; lwgaris@outlook.com (L.G.); ipike@bcchr.ca (I.P.); 3 City of Surrey Fire Service, Surrey, BC V3W 4P1, Canada; scmorris@surrey.ca (S.M.); chris.biantoro@surrey.ca (C.B.); 4 UWA Law School, The University of Western Australia, M253, 35 Stirling Highway, Crawley, WA 6009, Australia; joe.clare@uwa.edu.au; 5 Department of Security, Fire, and Emergency Management, John Jay College of Criminal Justice of the City University of New York, 524 West 59th Street, New York, NY 10019, USA; cjennings@manitouinc.com; 6 School of Culture, Media, and Society, The University of the Fraser Valley, Abbotsford, BC V2S 7M8, Canada; 7 Department of Pediatrics, Faculty of Medicine, The University of British Columbia, Vancouver, BC V6H 3V4, Canada

**Keywords:** residential fires, risk management, intervention strategies, longitudinal study, injury prevention

## Abstract

(1) Background: Residential fires represent the third leading cause of unintentional injuries globally. This study aims to offer an overview and a longitudinal evaluation of the HomeSafe program implemented in Surrey in 2008 and to assess its effectiveness in mitigating fire-related outcomes. (2) Methods: Data were collected over a 12-year period (2008–2019). Assessed outcomes comprised frequency of fire incidents, residential fires, casualties, functioning smoke alarms, and contained fires. The effectiveness of each initiative was determined by comparing the specific intervention group outcome and the city-wide outcome to the pre-intervention period. (3) Results: This study targeted 120,349 households. HomeSafe achieved overwhelming success in decreasing fire rates (−80%), increasing functioning smoke alarms (+60%), increasing the percentage of contained fires (+94%), and decreasing fire casualties (−40%). The study findings confirm that the three most effective HomeSafe initiatives were firefighters’ visits of households, inspections and installations of smoke alarms, and verifications of fire crew alarms at fire incidents. Some initiatives were less successful, including post-door hangers (+12%) and package distribution (+15%). (4) Conclusions: The HomeSafe program effectively decreased the occurrence and magnitude of residential fires. Lessons learned should be transferred to similar contexts to implement an evidence-based, consistent, and systematic approach to sustainable fire prevention initiatives.

## 1. Introduction

Fire-related mortality and morbidity represent the third leading causes of residential unintentional injuries globally [1,2,3]. Fires contribute to the global burden of disease, accounting for approximately 9 million injuries, 120,000 deaths, and 7.46 million Disability-Adjusted Life Years (DALYs) lost annually [2,4]. These estimates of fire-related fatal and non-fatal injuries may be underrated due to inconsistent and incomplete reporting of incidents, particularly in low- and middle-income countries [5]. Further to their high casualty rates, fires impose considerable economic loss, including nearly USD 8.6 billion in the US in 2020 [6]. Similar estimates of fire-related human and economic burdens emerge from Australia and Canada [7,8].

Residential fires remain a major health concern, accounting for a high proportion of injuries and deaths globally [5]. The existing literature has examined several factors associated with residential fires and their health outcomes, particularly those related to population characteristics and residential structures. Risk factors include low socio-economic status, residing in older buildings, male sex, age below 6 years of age or over 64, unemployment, lower levels of education attainment, consuming alcohol, and smoking [1,9]. Populations with unique characteristics and vulnerabilities tend to sustain an increased risk of fire-related morbidity and mortality [10,11]. Identifying and targeting high-risk groups within a population is critical to developing tailored fire safety messaging and smoke alarm installations, and, consequently, improving public safety behaviors and practices to reduce occurrences of fire incidents [12]. 

To mitigate the burden of residential fires, several intervention strategies and best practices have been adopted and implemented globally [13]. Evidence consistently indicates the relevance of effective community-based fire safety initiatives in curtailing the prevalence of residential fires and related injuries and fatalities [14,15,16,17]. Interventions, such as home visits, provision of fire safety educational messages, inspections, and installation of smoke alarms, are particularly effective in decreasing the frequency of fire incidents and casualty rates [14,18]. Further to the environmental safety modifications, the effectiveness and sustainability of fire prevention programs are strongly influenced by human cognitive and psychological factors. The ‘knowing–doing gap’ (uptake), which refers to the challenge of translating knowledge into effective actions in fire prevention, and the ‘wear-off effect’ (decay), which signifies the decreased effectiveness of interventions over time due to the lack of maintenance, constitute two factors that significantly influence the long-term effect of any implemented safety intervention [19]. To be mindful of the cycle of uptake and decay requires continuous monitoring and retreating with the intervention when decay or adverse effects are observed. Acknowledging the apparent effect of these factors on the attenuation of gained knowledge is critical and calls for timely refreshment to retain the acquired fire safety behaviors and practices over long time periods. 

Inspired by successful fire prevention programs globally [17], the ‘HomeSafe’ initiative was implemented in the city of Surrey, British Columbia in 2008 with the aim of decreasing the occurrence and impact of residential fires. An initial needs assessment was conducted prior to HomeSafe implementation, which served primarily to identify populations at high risk and to support program prioritization based on population characteristics, including young children, older adults, single parents, and individuals with low socio-economic status. Preliminary data revealed a steady increase in fire rates from 1988 to 2007 (80–88 fires per year per 100,000 population), with the majority being residential fires (75.5%) mainly occurring in single-family dwellings (87.5%) and caused by cooking (39.9%) and open flames (17%) [20] The assessment further showed the low prevalence of smoke alarm ownership and function, as nearly 36.5% of households had no smoke alarm installed and 49.5% had malfunctioning smoke alarms. Further to fire-related property damages, human suffering was substantial; nearly one in ten residential fires resulted in injuries (9.8%) and fatalities (0.8%). A considerable proportion of the reported injuries (58.2%) and deaths (83.3%) occurred in households that lacked a functioning smoke alarm. The needs assessment provided data-driven evidence to support the implementation of the proposed HomeSafe program. The various approaches and initiatives adopted within the HomeSafe program were inspired by the latest research on socio-economic factors and fire risks [21]. 

Initial evaluation results demonstrated positive effects of this program over the first few years [22]. Building on these findings, the main objective of this study is to provide a summary understanding of the fire prevention initiatives included within the HomeSafe program over 12 years in the city of Surrey, British Columbia. More importantly, this study provides a longitudinal analysis of the effectiveness of various initiatives within the HomeSafe program and evaluates their impacts on reducing the frequency and severity of residential fires and mitigating fire casualties. To achieve these objectives, this study addresses two main research questions: (1) What were the key fire prevention initiatives implemented within the HomeSafe program in the city of Surrey during the study period, and how effective were these initiatives in reducing the frequency and severity of residential fires? (2) What impact did these initiatives have on mitigating fire-related casualties, and what are the implications of the effective outreach and longevity of these initiatives for fire prevention strategies? The implications of the effective HomeSafe outreach initiatives and the longevity of their successful implementations are discussed in this paper, particularly with respect to the importance of ongoing, positive, collegial relationships between firefighters, fire service managers, and city management more broadly.

## 2. Materials and Methods

### 2.1. Study Design and Tool

**Figure 1:**
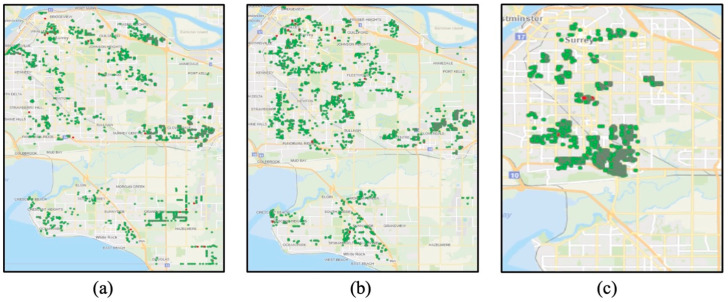
Area distribution in various neighborhoods within the city of Surrey of temporal cohorts: (**a**) cohorts 1–7, (**b**) cohorts 8–14, and (**c**) cohorts 15–18.

**Figure 2:**
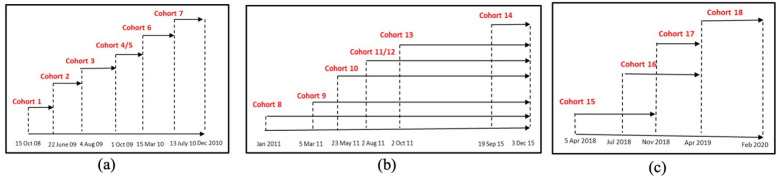
Distribution and timeline for cohorts 1–18: (**a**) cohorts 1–7, (**b**) cohorts 8–14, and (**c**) cohorts 15–18.

A longitudinal study was conducted to assess the effectiveness of HomeSafe fire prevention initiatives implemented in high-risk fire areas within the city of Surrey, Canada for the 12-year period (2008 to 2019). To identify high-risk fire areas, information was used from the 2016 census of the Canadian population (i.e., population characteristics and fire risk predisposition) and the National Fire Information Database (NFID, historical fire occurrence) to calculate a risk score for each Dissemination Area (DA, i.e., high-, medium-, and low-risk areas; for further details on the fire risk index method, please refer to Appendix A). The focus for this study was single-family homes. Apartments differ in the number of residences they encompass, and there is often an assertion that regular system testing and compliance with the National Fire Safety regulations is adhered to. However, improving apartments is different than improving a single-family residence due to the high expectation associated with the multiple units present in apartments and also the types of maintenance necessary to maintain these housing units as safe for residents. Smoke detectors used in all of this study’s phases were connected via wireless links where applicable. Typically, the fire alarms are connected to a central monitoring station at the fire department in order to automatically notify on-duty fire crews in the event of a fire. However, a smoke alarm is only a local alert, and it would be necessary to alert the fire department via telephone. In order to optimize the effectiveness of the HomeSafe program, areas with a higher likelihood of fire incidents were identified and selected as the primary focus. Within these identified regions, specific street addresses were sampled to create a targeted distribution of areas for HomeSafe visits. To minimize unnecessary travel, the selected addresses were grouped geographically, ensuring that on-duty firefighters could cover multiple locations efficiently. Participation in the HomeSafe program was voluntary. The Surrey Fire Service adopted a multi-pronged approach to implement three fire safety initiatives as part of the HomeSafe program:

HomeSafe1 (2008–2010): through this initiative, the Surrey Fire Service generated high-risk target areas and grouped them into cohorts (1–7) by geographic proximity (Figure 1a). Cohorts were targeted through door-to-door visits to promote fire safety education via safety packages (Appendix A) and free smoke alarm inspection and/or installation (Figure 2a). Moreover, residents were invited to request home safety inspections and installation of a smoke alarm. In total, 193 residential homes submitted requests (Table 1). A total of 18,473 high-risk residential dwellings were targeted in the HomeSafe1 initiative.

HomeSafe2 (2011–2017): Through this initiative, seven new cohorts (cohorts 8–14) in high-risk areas were targeted via door-to-door visits by fire crews (Figure 1b). HomeSafe2 promoted fire safety awareness and smoke alarm installation and/or inspection at multiple city events, such as the property tax payment lineup (15,666 households reached from 2015 to 2017), Food Bank (3500 households reached from 2015 to 2017), and the British Columbia Smoke Alarm Movement (since 2012, 41,000 smoke alarms distributed across BC and more than 20,000 smoke alarms distributed to seniors with limited mobility and other chronic diseases). A total of 21,408 households were identified as high-risk and targeted (Figure 2b). In 2015, Surrey firefighters and volunteers utilized a three-stage treatment program to educate residents with fire safety messages:

Treatment 1: Firefighters conducted door-to-door visits for 500 households within cohort 14.

Treatment 2: Home visits were made to 4600 high-risk residents within cohort 13.

Treatment 3: Informational door hangers were distributed to 8740 residential properties identified via spatial clustering and outlier analysis.

HomeSafe3 (2018–2019): Through this initiative, community volunteers engaged in conducting door-to-door visits and telemarketing (phone calls) to 4 new cohorts (cohorts 15–18) in high-risk areas (Figure 1c) using GIS tools for efficiently promoting fire safety and encouraging smoke alarm installation and inspection (Figure 2c). Fire crews continued to promote fire safety during attendance at residential incidents. During this period, 4856 high-risk households were targeted via door-to-door visits by volunteers, in addition to the 9000 households reached via telemarketing.

### 2.2. Data Collection and Analysis

Data were collected from the HomeSafe initiatives and the Surrey Fire Service for the period from 2008 to 2019. Data prior to HomeSafe were collected using the British Columbia Office of the Fire Commissioner (OFC) and the Surrey Fire Service.

To assess the effectiveness of the HomeSafe initiatives, the frequency and severity of residential fires experienced before and after implementing the intervention were compared. To measure the impact of the initiatives, data were compared to residential fire data captured by the British Columbia OFC in the 3 years prior to the implementation of HomesSafe1 (2005–2007).

For HomeSafe1, a randomized high-risk cluster control method was adopted to assess fire-related outcomes between the intervention cohorts and randomized control cohorts with equivalent fire risk but with no fire prevention intervention. HomeSafe1 rates were compared between the targeted cohorts and the control group. For HomeSafe2 and HomeSafe3, an Analysis of Variance (ANOVA) was conducted to determine the significance of the between-group effect on fire rates in home visits.

The effectiveness of each implemented initiative was determined by comparing the specific intervention group outcome and city-wide outcome during each HomeSafe initiative period to the pre-intervention period. The investigated intervention group outcomes included: (1) residential fire rates (per 1000 dwellings), (2) proportion of functional smoke alarms, and (3) proportion of fires confined to the object of origin (as a proxy to fire severity). The investigated city-wide measures included: (1) fire rates (per 100,000 residential structures), (2) fire-related injury and death rates (per 100,000 population), (3) proportion of functioning smoke alarms at the time of residential fire incidents, and (4) proportion of fires confined to the room of origin. Further comparisons included: (1) total number of requested smoke alarm inspections and/or installations, (2) smoke alarm verifications at incidents, and (3) households reached at various events. To prevent any discrepancies, abandoned properties were excluded from the evaluation as they introduce unique challenges and require interventions beyond the scope of this study [23]. This exclusion allows for the effective monitoring of the outcomes of HomeSafe interventions.

## 3. Results

**Figure 3:**
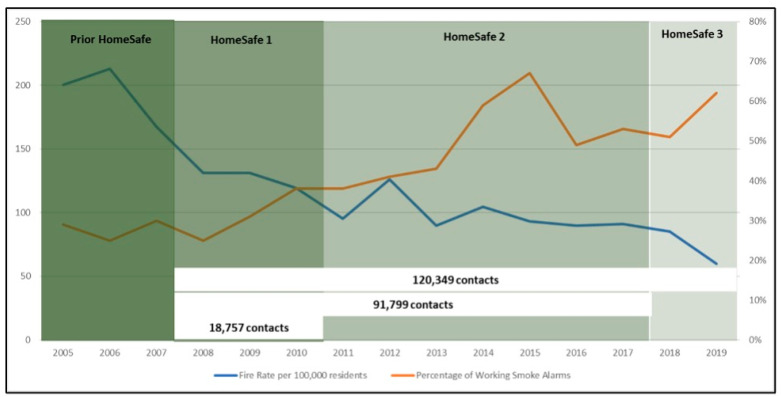
Change in the residential fire rate (per 100,000 residents) and functional smoke alarms (%) during the 4 periods.

A total of 120,349 households participated in the 12-year period of the HomeSafe program (18,757 in HomeSafe1-HS1, 91,799 in HomeSafe2-HS2, and 9793 in HomeSafe3-HS3, Figure 3).

The implemented HomeSafe initiatives yielded a substantial increase in functioning smoke alarms and a significant decrease in residential fire occurrences and fatalities. This was achieved through cohort visits conducted by firefighters, inspection and installation of smoke alarms conducted upon request, and validation of smoke alarms by fire crews during incidents. Nonetheless, a reverse pattern was reported in relation to the interventions related to distributing information packages and door-to-door hangers, which failed to show any effectiveness in reducing fire rates.

The HomeSafe1 (HS1) initiative led to a 91% reduction in residential fires, with a 50% reduction still apparent 5 years post-intervention. Among the 91 smoke alarm functioning verifications completed by fire crews at residential fire incidents, only 2.2% had post-visit fires (Table 1). Pre- and post-visits showed a 64% decrease in fire rates in the intervention group and a 14.6% decrease in the control group.

The HomeSafe2 (HS2) initiative demonstrated a positive impact on reducing residential fire rates by 75% with smoke alarm installations by request, and a reduction of 66% where fire crew smoke alarm verifications took place at incidents. There was an increasing trend in the percentage of functional smoke alarms, 61.5%, at locations where smoke alarms were installed, as well as an increase of 6.1% where crews verified the presence of functioning smoke alarms (Table 1). In contrast, door hangers and drop-off information packages without the presence of a fire representative did not yield the expected results. A negative impact manifested in a proportional decrease in functional smoke alarms (−46.4% and −16.5%, respectively), and a relative increase in residential fires (+12% and +15%, respectively; Table 1).

The HomeSafe3 (HS3) initiative revealed a favorable impact on increasing the rate of functioning smoke alarms from 35% to 100% through home visits by volunteers, as well as the proportion of fires confined to the object of origin (67%). Additionally, this initiative successfully reduced fire rates by 33% for smoke alarm installation upon request and by 87% for verification of smoke alarms by the fire crew at incidents (Table 1).

### 3.1. Intervention Group Outcomes

Over the period of the HomeSafe1 initiative, intervention groups sustained a residential fire each 97.3 and 193.1 days before and after the intervention, respectively, compared to the control group, which experienced a residential fire every 64.1 and 68.8 days pre- and post-intervention, respectively [15] (Table 2).

**Figure 4:**
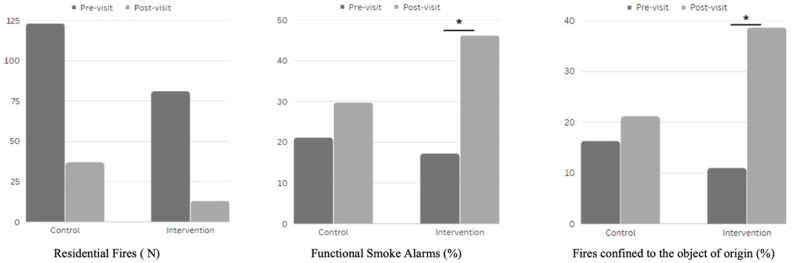
Change in post-intervention fire rates between the control and intervention groups for residential fires, functioning smoke alarms, and fires confined to the object of origin. * Significant increase between pre- and post-visit within group (p-value≤0.05).

Evaluating the severity of residential fires during HomeSafe1 showed that the proportion of functioning smoke alarms and fires confined to the object of origin significantly increased pre- and post-visit in the intervention group (X^2^ (1, N = 94) = 5.57, *p*-value < 0.05 and X^2^ (1, N = 94) = 6.61, *p*-value < 0.02, respectively). The ANOVA demonstrated the impact of in-home visits on the fire rates, resulting in a significant between-group effect F (1, 12) = 8.31, (*p*-value < 0.02). Post hoc analysis showed substantial differences in post-intervention fire rates between the intervention and control groups F (1, 12) = 6.56, (*p*-value < 0.03) (Figure 4 and Table 3).

During HomeSafe2, a 57% decrease in residential fires was reported in the intervention cohorts following the door-to-door visits, in addition to a noticeable rise in the proportion of functional smoke alarms (23%) and residential fires contained within the object of origin (12%) (Table 4).

During HomeSafe3, the residential fire rate in cohorts 15–18 decreased by 80% to reach 0.31 fires per 1000 occupied properties, while the percentage of functional smoke alarms increased by 183% and the residential fires confined to the object of origin after the visit decreased by 33% (Table 5).

### 3.2. City-Wide Outcomes

**Figure 5:**
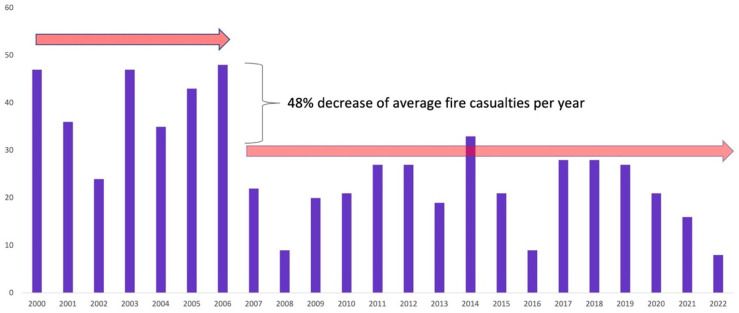
Residential fires in Surrey after HomeSafe intervention.

During the initial three years of the HomeSafe1 program, successful city-wide outcomes were illustrated by a 34% reduction in residential fires and a 58% reduction in the fire casualty rate, while the number of residential properties with functional smoke alarms increased by 11% and the number of residential fires contained to the object of origin increased by nearly 54% (Figure 5).

During HomeSafe2, Surrey City sustained a 22% decrease in residential fires, a 39% increase in functioning smoke alarms (66.75 increase in 2015 alone), a 20% decrease in fires contained within the room of origin, and a 24% increase in the casualty rate (Table 6).

In the HomeSafe3 period, residential fire rates in the city of Surrey continued to decrease to reach a 26% reduction since HS2 and a 43% reduction since the onset of the HomeSafe1 initiative in 2008. The proportion of functional smoke alarms increased by 130% to reach 56%, with 43% of residential fires contained within the object of origin (Table 6). The casualty rate increased by 9% on average since the end of HS2. However, by the end of 2019, there was a drop of 0.4 in the number of casualties per 100,000 population (Table 6).

## 4. Discussion

This study presents the outcomes of the HomeSafe Program over a period of 12 years and demonstrates the effectiveness of implemented fire interventions in decreasing the occurrence and the impact of residential fires, enhancing fire safety measures, and reducing fire-related injuries and deaths.

Throughout the implementation period, HomeSafe achieved considerable success in decreasing fire rates within the city of Surrey by 80%, increasing the presence of operational smoke alarms in residential homes by 60%, improving fire confinement to the initial source by 94%, and, more importantly, decreasing fire casualties by more than 40%. The study findings confirm that the three most effective HomeSafe initiatives were the household visits conducted by on-duty firefighters, smoke alarm inspections and installations upon request, and the validation of smoke alarms by fire crews during fire incidents.

Despite the overall success of the HomeSafe program, data showed that some initiatives were more effective than others for the measured outcomes. For instance, outreach initiatives, such as information package drop-offs and door hangers, which lack direct personal contact and informational transactions with household residents, have proven to be less effective at ameliorating fire-related outcomes. As for telemarketing outreach, the data set of contact numbers available was somewhat random and not necessarily aligned with the targeted risk criteria. The post-outreach analysis demonstrated the absence of fire incidents reported in the contacted properties pre- or post-implementation of the initiative. Aligning with existing studies, the evidence from this study further demonstrates that door-to-door visits by firefighters or public safety volunteers served to enhance residents’ safety knowledge through interactive and engaging educational information and to ensure functioning fire alarms were installed in targeted households [24,25]. This approach was particularly effective in significantly protecting communities against residential fires. Several studies have underscored the importance of engaging with household members through direct face-to-face personal contact and hands-on training for enhancing individuals’ acquisition of safety knowledge and the adoption of fire safety behaviors and practices [26,27,28].

Aligning with the international literature, this study confirms that households with functioning fire alarms reported remarkably low rates of fire-related injuries and deaths [29]. Installing and maintaining functional smoke alarms at residential properties is one of the prominent residential fire prevention strategies and key to sustainably securing fire safety over time [28,30,31]. Moreover, this study shows that targeting high-risk households through an initial assessment and mapping using geographic information systems (GIS) [14,15,30,32] was critical to substantially reducing fire casualties among vulnerable populations. Selected household characteristics tend to increase individuals’ vulnerability to and risk of fires, including households with children aged less than 6 or older adults aged more than 64, single parents, residents who are frequent movers, residents with low income or unemployment status, those residing in older buildings, individuals with disabilities, and immigrants [33,34]. Moreover, tailored and culturally sensitive fire safety interventions were shown to effectively reduce fire-related morbidity and mortality, particularly among populations with unique demographic characteristics [35,36,37,38].

The HomeSafe program offers opportunities for lessons learned and presents a series of recommendations for future fire prevention programs, including:

### 4.1. Engaging Community and Government

Community engagement and government support are key to enhancing safety and reducing residential fires [14,15]. Active commitment from the municipal government to fund and implement fire awareness programs, promote smoke alarm installation, and enforce fire safety measures coupled with fire service support and community engagement with fire prevention programs represent a vital step toward a safer community.

### 4.2. Focusing on Successful Initiatives

As demonstrated in this study, variation in the success of the HomeSafe initiatives was clearly highlighted. Door visits conducted by firefighters, smoke alarm inspection and installation upon request, as well as fire crew validation of smoke alarms at fire incidents were proven effective in raising awareness about fire safety, advising on potential hazards at home, and, consequently, reducing the occurrence and severity of fires. However, initiatives that lacked social interaction opportunities, such as personal contact and dialogue with recipients, were less successful. It is critical to identify which initiative has the potential to succeed and to invest efforts into its proper implementation.

### 4.3. Targeting High-Risk Populations

To ensure an effective fire safety program, the intervention should target high-risk populations and should regularly update and map the higher-risk neighborhoods (Figures S1 and S2). This approach will increase the likelihood that the program is directed toward the targeted population. Hence, it is critical that the intervention program is supported by a comprehensive data repository of updated population demographics and information related to city planning and development to ensure constant monitoring. As the fire safety program evolves, it is important to continuously assess population characteristics within cities and identify high-risk residents to ensure successful and sustainable interventions.

### 4.4. Conducting Longitudinal Assessment of Implemented Initiative

Ongoing monitoring and evaluation of the fire interventions are crucial to ensure the long-term success of the initiative [14]. To maintain the sustainability of the fire intervention, a longitudinal and evidence-based assessment, followed by re-assessment, is needed to ensure the detection of any changes in the progression of the implemented initiatives and measures and adapt accordingly.

### 4.5. Ensuring Sustainability and Long-Term Programs

Implementing a large-scale program that aims to reach a sizeable proportion of the targeted population can lead to a sustainable decrease in fire-related injury and death rates over decades.

To enhance program sustainability, it is important to tailor the intervention based on new protocols associated with any surging pandemic and to address the challenge of maintaining effective program operation while prioritizing the health and safety of crews, volunteers, and residents. Future fire intervention programs should take into consideration the ‘knowing–doing gap’ and the ‘wear-off’ effect [19] to ensure the long-term impact of the intervention program on enhancing individuals’ knowledge and practicing safety behavior over time. The sustainability and ongoing effectiveness of this work would also not have been possible without the commitment shown from city management, fire service management, the firefighter union, the firefighters, and a range of other service providers throughout the municipality. The program’s success was not an accident. It depended on participation from key stakeholders with a shared vision for safety. Continuing to implement evidence-based fire prevention across changes in leadership at the city level, fire service level, and union level is a success in its own right. The results of this success are clearly demonstrated in the longitudinal reductions in fire loss and casualty documented through this paper.

### 4.6. Highlighting the Importance of Management

One of the major contributing factors to the success of the HomeSafe program is the adoption of a systematic and coherent approach for conducting fire safety campaigns as well as validation and installation of smoke alarms. This approach has shown positive impacts on the visited properties and the entire city. The success of the program reinforces existing research and emphasizes the significance of evidence-based, systematic, and continuing dissemination of fire safety information to residences together with smoke alarm testing and installation services. The mutual benefits of reducing community residential fires and improving firefighters’ jobs with less exposure to fires, the produced smoke’s toxic chemicals, and the associated risk of contracting occupational-related cancers led to more sustainable and consistently monitored fire safety initiatives across the community over time.

### 4.7. Benchmarking and Informing Evidence-Based Policies

Synthesized findings from the HomeSafe program can be adopted by policy makers and key stakeholders to inform evidence-based safety regulations and data-driven decisions on resource allocation and fire safety program implementation. Further to supporting policy makers’ decisions, findings from the HomeSafe program can be adopted to benchmark the effectiveness of various fire prevention programs in other regions and to use HomeSafe as a model program to evaluate existing programs and refine and make changes to improve outcomes.

The long-term, sustained commitment to the program across the Surrey Fire Service is exceptional. The mutual understanding between fire management and firefighters has led to a cultural shift of incorporating the activities of HomeSafe into regular daily duties. By verifying a working smoke alarm at every residential opportunity, the program becomes sustainable on an ongoing basis. One of the distinguishing features of this program can be explained by the literature on government innovation. While the definition of innovation remains contested [39], this program certainly qualifies as being innovative in any practical sense.

Using a framework of key factors for innovation, it is apparent that this success was due to several organizational, managerial, and cultural conditions. In Table 7, we account for these factors and explain their importance to the efforts. This framework may offer some explanatory insights into the underlying components of this program’s success.

This study has some strengths and limitations. The systematic and consistent approach adopted to implement the fire intervention represents the major strength of this study. The longitudinal aspect of the HomeSafe initiative, with the fire safety campaigns and smoke alarm testing and installations, constitutes a key strength of the HomeSafe program. This study faces a few challenges and incurs some limitations. One of the main limitations of the HomeSafe initiative is the ability to map neighborhoods that align with the profile of the targeted population while taking into consideration the dynamic environment of the city based on its organization, development, and residents’ socio-economic status. The lack of recent and updated population demographic data hindered the precise identification of high-risk areas for HomeSafe targeting. However, a possible solution for properly identifying high-risk areas would be the implementation of a nearly real-time monitoring system for each initiative and merging it with an up-to-date database of population demographics, as well as city organization and development. Additionally, within each HomeSafe initiative, residents’ demographic data could be collected whenever social interaction with residents is possible. This would further enhance the overall data collection process. Another limitation refers to the spread of the coronavirus pandemic in 2019, which coincided with the implementation of HomeSafe3.

This posed a new challenge in maintaining the effective operation of the HomeSafe program without risking the health and safety of residents, fire crews, and community volunteers. Restricted social interaction among residents hindered the promotion of fire safety campaigns for at-risk residents. Existing fire initiatives should adapt to and accommodate the spreading of the pandemic while aligning with protocols and measures for the well-being of individuals. Nonetheless, these obstacles created opportunities for program enhancements that can be explored in future work. Finally, the effectiveness and outcomes of the tax lineup and Surrey Food Bank initiatives were impeded by the challenges encountered in collecting property information during these events.

## 5. Conclusions

This longitudinal study assessed the effectiveness of several fire safety initiatives implemented through the HomeSafe program and their impacts on enhancing fire safety and reducing fire-related outcomes. The HomeSafe program provides clear evidence on the effectiveness of targeted interactional educational and environmental modification interventions as part of an overall community risk reduction initiative. Future opportunities to interact with the targeted social groups for the promotion of the program and safety outcomes may assist with expanding the message to those who are not picked up in the program delivery areas.

## Figures and Tables

**Table 1:** Change in residential fire trends pre- and post-interventions (HomeSafe1-HS1, HomeSafe2-HS2, and HomeSafe3-HS3).

Cohort			Requested Inspection and/or Installation (HS1)	Smoke Alarms Verified at Incidents (HS1)	Requested Inspection and/or Installation (HS2)	Door Hangers (HS2)	Packages (HS2)	Smoke Alarms Verified at Incidents (HS2)	Requested Inspection and/or Installation (HS3)	Smoke Alarms Verified at Incidents (HS3)
Addresses (N)			193	91	3284	8740	4630	15,814	769	13,175
Annual Fire Rate (per 1000 dwellings)	Pre-Visit		5.2	N/A	3.2	2	2.05	1.77	1.95	1.1
	Post-Visit		0.47	1.8	0.79	2.24	2.35	0.61	1.3	0.14
	Rate Change (%)		−91	--	−75	+12	+15	−66	−33	−87
	Residential Fires Post-Visit (%)	1 Yr.	0	0	19.2	21.6	22.4	40.6	100	89.5
		3 Yrs.	0	50	65.4	78.4	67.3	81.4	100	100
		5 Yrs.	50	100	100	100	100	98	100	100
		10 Yrs.	100	100	100	100	100	100	100	100
Functional Smoke Alarms (%)	Pre-Visit		50	N/A	38.1	65.7	68.4	58.9	33.3	69
	Post-Visit		0	100	61.5	35.2	57.1	62.5	50	73.7
	Rate Change (%)		−100	--	+61.5	−46.4	−16.5	+6.1	+50	+6.8
Fires confined to the object of origin (%)	Pre-Visit		50	N/A	4.8	14.3	0	16.1	0	0
	Post-Visit		0	0	7.7	2.3	4	8.3	0	5.3
	Rate Change (%)		−100	--	+62	−84	N/A	−48	N/A	N/A

**Table 2:** Fire rate per 1000 dwellings annually for both the intervention and control groups. * Significant difference in fire rate between groups post-intervention (*p*-value ≤ 0.05).

Cohort	Addresses (N)	Time Pre-Visit (Year)	Time Post-Visit (Year)	Annual Fire Rate for Intervention Group (per 1000 Dwellings)		Annual Fire Rate for Control Group (per 1000 Dwellings)	
				Pre-Visit	Post-Visit	Pre-Visit	Post-Visit
1	2747	2	2.07	2.18	1.23	3.64	3.34
2	2716	2.68	1.38	1.23	0	2.61	1.33
3	2690	2.8	1.27	1.19	0.59	2.65	1.47
4	2627	2.99	1.08	0.76	0.71	2.04	2.12
5	2803	3.41	0.65	1.05	1.09	1.99	0
6	2407	3.74	0.33	0.56	0	1	1.27
7	2483	3.97	0.1	3.04	0	2.03	4.08
Total	18,473	3.09	0.98	1.43	0.52 *	2.28	1.95

**Table 3:** Severity of residential fires when confined to the object of origin and the presence of working smoke alarms pre- and post-visits for intervention and control groups; * (*p*-value ≤ 0.0.5).

Group	Timing	Residential Fires (N)	Functional Smoke Alarms (%)	Fires Confined to the Object of Origin (%)
Control	Pre-Visit	123	21.1	16.3
	Post-Visit	37	29.7	21.16
Intervention	Pre-Visit	81	17.2	11
	Post-Visit	13	46.2 *	38.6 *

**Table 4:** Fire rate per 1000 properties, presence of functional smoke alarms (%), frequency and severity of fires confined to the object of origin (%), and fires post-intervention (%) within 1, 3, 5, and 10 years.

Cohort	Addresses (N)	Fire Rate (per 1000 Occupied Properties)			Functional Smoke Alarms (%)			Residential Fires Confined within the Object of Origin (%)			Residential Fires Post-Intervention (%)			
		Pre-Intrv	Post-Intrv	Rate Change (%)	Pre-Intrv	Post-Intrv	Rate Change (%)	Pre-Intrv	Post-Intrv	Rate Change (%)	1 Yr.	3 Yrs.	5 Yrs.	10 Yrs.
8	2789	3.76	0.8	−79	33.3	48	44	71.4	92	29	24	48.1	64	100
9	2672	2.25	0.82	−64	25	61.5	146	83.3	100	20	11.5	42.3	61.5	100
10	2772	2.17	1.12	−48	33.3	48.3	45	91.7	93.1	2	20.7	48.3	76	100
11	1918	1.82	0.87	−52	57.1	60	5	100	90	−10	5	30	60	100
12	2359	4.03	1.28	−68	36.8	56	52	68.4	91.2	33	35.3	73.5	79.4	100
13	8387	3.3	1.27	−62	58.2	56.7	−3	92.7	97.6	5	29.1	76.4	93.7	100
14	511	0	0	N/A	0	0	--	0	0	--	0	0	0	0
Total	21,408	2.94	1.26	−57	45.2	55.6	23	84.9	95.4	12	25	63.2	81.2	100

**Table 5:** Fire rate per 1000 dwellings per year, the severity of residential fires in relation to being confined to the object of origin (%), and presence of functional smoke alarms (%) pre- and post-intervention for cohorts 15 to 18.

Cohort	Addresses (N)	Fire Rate (per 1000 Occupied Properties)			Functional Smoke Alarms (%)			Residential Fires Confined within the Object of Origin (%)			Residential Fires Post-Intervention (%)			
		Pre-Intrv	Post-Intrv	Rate Change (%)	Pre-Intrv	Post-Intrv	Rate Change (%)	Pre-Intrv	Post-Intrv	Rate Change (%)	1 Yr.	3 Yrs.	5 Yrs.	10 Yrs.
15	833	3.0	0.54	−82	67	100	50	100	0	−100	100	100	100	100
16	1491	1.01	1.06	5	0	1001	100	100	100	0	66.7	100	100	100
17	906	1.1	0	−100	50	N/A	N/A	100	N/A	N/A	N/A	N/A	N/A	N/A
18	1626	1.54	0	−100	17	N/A	N/A	100	N/A	N/A	N/A	N/A	N/A	N/A
Total	4856	1.54	0.31	−80	35.3	100	183	100	67	−33	75	100	100	100

**Table 6:** The city-wide impact of HomeSafe initiatives by comparing residential fire-related outcomes pre- and post-HS1, HS2, and HS3.

Timing	Residential Fire Rate (per 100,000 Residential Units)	Functional Smoke Alarms (%)	Fires Confined to the Room of Origin (%)	Casualty Rate (per 100,000 Population)
Pre-HS1	194	28	48	8.6
Post-HS1	127	31	54	3.6
Rate Change (%)	−34	+11	+12.5	−58
Pre-HS2	127	31	54	3.6
Post-HS2	98	43	43	4.4
Rate Change (%)	−22	+39	−20	+24
Pre-HS3	98	43	43	4.4
Post-HS3	72	56	43	4.8
Rate Change (%)	−26	+130	0	+9

**Table 7:** Key factors that influence the public sector innovation process [40].

	Actor(s)	Analysis
Governance and innovation	Chief and upper management value innovation	Surrey has pride in its reputation for problem solving
Sources of ideas for innovation	Management conducts a thorough search of the scholarly and best practice literature	Identify and partner with researchers and institutions
Innovation culture	Positive labor management climate; agency reputation for nurturing new ideas	Union committed to a community service focus; recognition of shared benefits (community and workforce) of preventive activity
Capabilities and tools	Availability of in-house analytic and evaluation expertise; agency partners with other organizations to expand capabilities	Surrey is unique in having a doctoral-level analyst on staff; the agency identifies opportunities for partnership, such as the use of volunteers, academic partners, and other city agencies
Objectives, outcomes, drivers, and obstacles	Ongoing adaptation of the program led by management	Sustained management attention and commitment

## Data Availability

Data will be available upon reasonable request from the corresponding author.

## Supplementary Materials

The following are available online at https://www.mdpi.com/article/10.3390/ijerph20146369/s1, Figure S1: Density of Fire Incidents over the last 5 years at the City of Surrey; Figure S2: Areas with Higher Risk of Population Characteristics across the City of Surrey.

## Appendix A. The Statistical Geomatics Centre Proposal for a Composite Index of Residential Fire Risk

The goal of this project is to identify fire high-risk areas based on the information from the 2016 census of the Canadian population and NFID (National Fire Information Database). These two data components will be used to calculate a risk score for each DA (Dissemination Area) along two dimensions: population residential fire risk predisposition (based on the selected population characteristics—census profile data) and historical fire occurrence (NFID data).

The project builds on elements of the residential fire high-risk areas identification methodology utilized by the Surrey Fire Service (Surrey, BC) and incorporates additional steps to produce a composite risk index at the DA level.

Risk scoring can be conducted at different risk sensitivity levels by selecting the top 10% percentile.

Data: NFID (National Fire Information Database) aggregated to the DA level and 2016 census of the population profile data at the DA level.

Population: All DAs are located in the province of British Columbia. The information from NFID is categorized to the residential structures under the following residential use classes: (34) residential (single detached); (35) duplex, three-plex, four-plex, semi-detached; and (37) camp site/RV park. This project excluded residences including condominiums, townhouse complexes, and apartments that regularly undergo mandatory annual inspection.

Methodology: The literature [15] has suggested using the following population characteristics as the risk factors to identify residential fire-prone areas:

1. Population under 6 years old;
2. Population over 64 years old;
3. Single-parent family;
4. Unemployment rate;
5. Mobile population.

Adjusting for the Canadian 2016 census data, the following population profile variables will be used:

1. Age groups and the population’s mean age—100% data—0 to 4 years, %;
2. Age groups and the population’s mean age—100% data—65 years and over, %;
3. Total number of census families in private households—100% data—total lone-parent families by sex of parent, %;
4. Unemployment rate, %;
5. Total—Mobility status 1 year ago—25% sample data—Movers, %.

Residential fire occurrence information will be derived from the NFID aggregated to DA data, with which it is possible to calculate the following information on residential fires:

1. Residential fire incident rate per 1000 residential structures or 1000 total population;
2. Proportion of functional smoke alarms during residential fire incidents;
3. Casualty rate per 1000 population.

### Steps for Calculating a Composite Fire Risk Index

The steps described below use the top 10% percentile as the threshold to identify DAs under fire risk.

1. StatCan provides census profile data as counts and as rates or percentages. For comparability between DAs and index building, the population characteristic data need to be measured as rates/percentages. The NFID aggregated data should also be used in the relative measures form, i.e., as either rates or percentages. In total, there are five indicators for the population fire risk predisposition and three for the fire occurrence dimension.
2. To make the geographical units of analysis (DAs) comparable across a total of eight indicators, the indicators need to be further standardized into z-scores. As these measures are all continuous data, this step can be accomplished using any standard statistical software or MS Excel. The z-score measures a value’s distance from the variable mean in standard deviations.
  The formula of the z-score is (Equation (A1)):
  (A1) $$Ζ=\left(\chi -\mu \right)/\sigma$$
  Ζ: standard score; χ: observed value; μ: sample mean; σ: sample standard deviation.
3. Using the z-score values and the probabilities distribution under the normal curve (Table A1), identify DAs that fall into the top 5% for each of the eight indicators. This step produces a new set of categorical variables (total of eight) with values of 1 = ’yes, a risk area’ and 0 = ’no, not a risk area,’ indicating whether a DA’s z-score on an indicator placed that DA in the top 5% percentile group.
4. The new categorical indicators are the input data for the composite index score for each of the two dimensions. Note from the previous step that if a DA has a z-score of 1.645 for an indicator, it receives a score of 1 for the new categorical variable for this indicator. In this step, the two summary variables are created: (1) population fire risk predisposition score, and (2) fire occurrence score via summation of the respective categorical indicator (1 = ’yes risk’ and 0 = ‘no risk’) variables. The maximum value of the population fire predisposition score is 5, as there are five population variables, and the fire occurrence score can have a maximum value of 3, as there are three variables pertaining to fire incidence data utilized in the analysis.
5. Two summary variables can then be juxtaposed to create a composite index as a result of the DAs’ positioning for each of the two dimensions. This can be accomplished through the classification of DAs into high-, medium-, and low-risk areas using the following schema (Table A2).
6. The composite index then becomes a product of these two classifications, where it is possible to distinguish the following twelve categories depending on the combination of the two standings (Table A3).

**Table A1:** Z-score.

Z-score	P (x > Z)
1.645	0.05
1.282	0.10
1.036	0.15
0.842	0.20

**Table A2:** Population Fire Risk Predisposition.

Population Fire Risk Predisposition		Fire Occurrence
High	High	3
Medium	Medium	2
Low	Low	1
Not a risk	Not a risk	0

**Table A3:** Population Fire Risk Predisposition.

	Population Fire Risk Predisposition				
Fire occurrence		High	Medium	Low	Not a risk
	High				
	Medium				
	Low				
	Not a risk				

This juxtaposition can be accomplished visually by creating a bivariate choropleth map, where each category is individually color coded. A bivariate map example: https://en.wikipedia.org/wiki/Multivariate_map#/media/File:Black_Hispanic_Bivariate_Map.png accessed on 1 November 2012.
