# Supplementary material for: Community Fire Risk Reduction: Longitudinal Assessment for HomeSafe Fire Prevention Program in Canada
Source: Int J Environ Res Public Health. 2023 Jul 15;20(14):6369. doi: 10.3390/ijerph20146369 (PMC10379429; doi:10.3390/ijerph20146369)

Supplementary Materials

Figure S1. Density of fire incidents over the last 5 years at the city of surrey.

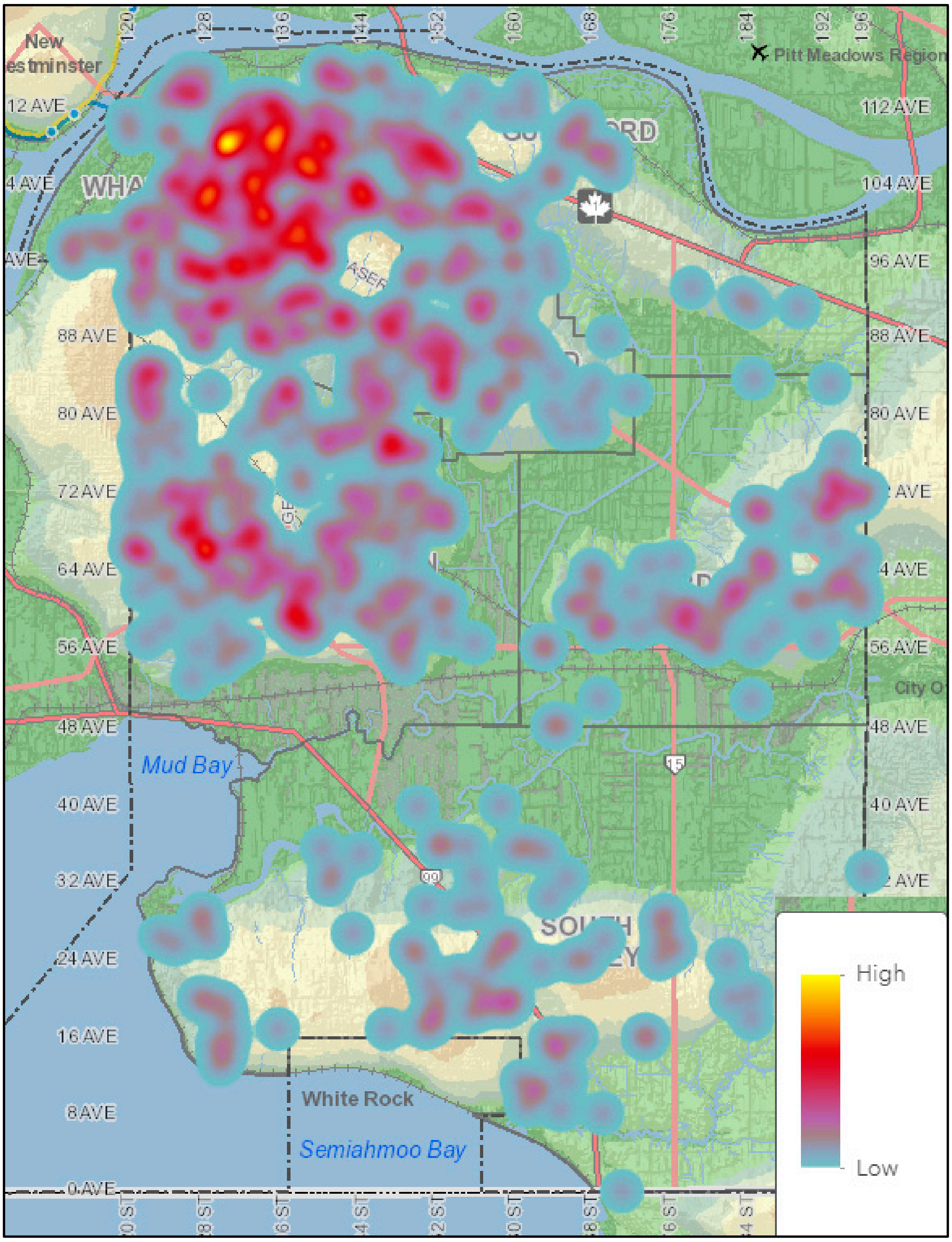

Figure S2. Areas with higher risk of population characteristics across the city of surrey.

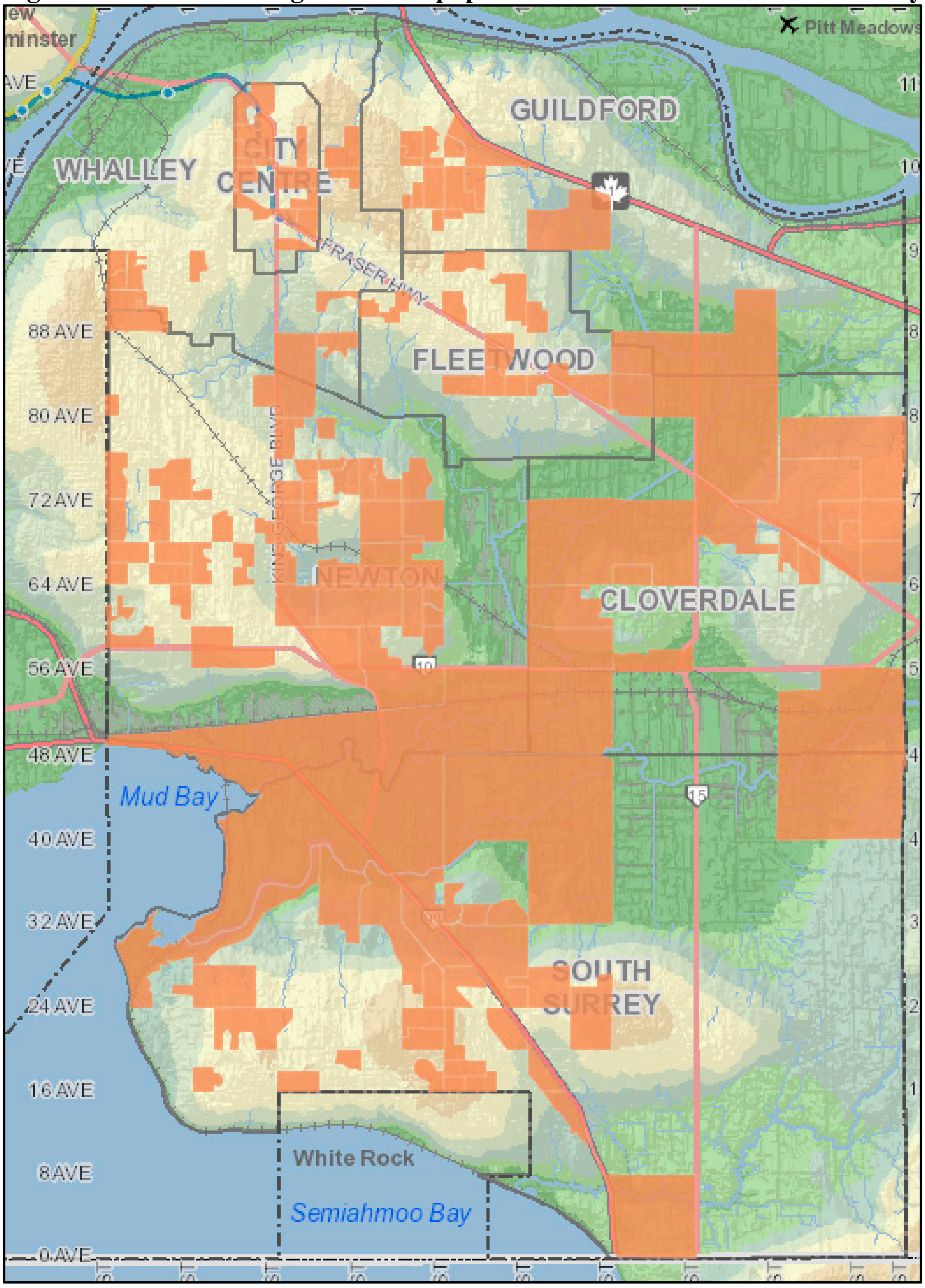

Supplement: Supplementary file 1 [file ijerph-20-06369-s001.zip › ijerph-2391681-supplementary.pdf]
